# Enhancing structural robustness of scale-free networks by information disturbance

**DOI:** 10.1038/s41598-017-07878-2

**Published:** 2017-08-08

**Authors:** Jun Wu, Suo-Yi Tan, Zhong Liu, Yue-Jin Tan, Xin Lu

**Affiliations:** College of Information System and Management, National University of Defense Technology, Changsha, Hunan 410073 P. R. China

## Abstract

Many real-world systems can be described by scale-free networks with power-law degree distributions. Scale-free networks show a “robust yet fragile” feature due to their heterogeneous degree distributions. We propose to enhance the structural robustness of scale-free networks against intentional attacks by changing the displayed network structure information rather than modifying the network structure itself. We first introduce a simple mathematical model for attack information and investigate the impact of attack information on the structural robustness of scale-free networks. Both analytical and numerical results show that decreasing slightly the attack information perfection by information disturbance can dramatically enhance the structural robustness of scale-free networks. Then we propose an optimization model of disturbance strategies in which the cost constraint is considered. We analyze the optimal disturbance strategies and show an interesting but counterintuitive finding that disturbing “poor nodes” with low degrees preferentially is more effective than disturbing “rich nodes” with high degrees preferentially. We demonstrate the efficiency of our method by comparison with edge addition method and validate the feasibility of our method in two real-world critical infrastructure networks.

## Introduction

Networks are everywhere. Examples include the Internet, metabolic networks, electric power grids, supply chains, urban road networks, the world trade web, among many others. In the past few years, the discoveries of small-world^1^ and scale-free^2^ properties has stimulated a great deal of interest in studying the underlying organizing principles of various complex networks. The efforts to develop a universal view of complex networks have created both excitement and confusion about the way in which knowledge of network structure can be used to understand, control, or design system behavior^3^. The investigation of complex networks has become an important area of multidisciplinary area involving physics, mathematics, operations research, biology, social sciences, informatics, and other theoretical and applied sciences^4–9^.

The functionality of complex networks relies on their structural robustness, i.e. the ability to retain its connectivity when a portion of their nodes or edges is removed^10, 11^. For example, modern society is dependent on its critical infrastructure networks: communication, electrical power, rail, and fuel distribution networks. Failure of any of these critical infrastructure networks can bring the ordinary activities of work and recreation to a standstill^12–14^. Terrorist attacks on transportation networks have traumatized modern societies. With a single blast, it has become possible to paralyze airline traffic, electric power supply, ground transportation or Internet communication. Other examples of structural robustness arise in nature, such as the robustness of food webs to biodiversity loss^15, 16^.

Because of its broad applications, the structural robustness of complex networks has received increasing attention, especially from the original work by Albert *et al*.^17^. They introduced two attack strategies, i.e., random failure and intentional attack. Albert *et al*. suggested that scale-free networks characterized by a highly heterogeneous degree distribution are robust against random failure but are very fragile against intentional attack. This property is referred to as the “robust yet fragile” feature or the Achilles’ heel of scale-free networks by Doyle *et al*.^18, 19^. Because of the ubiquity of scale-free networks in natural and man-made systems, the structural robustness of scale-free networks has been of great interest since the discovery of the scale-free property.

Let’s recall the “robust yet fragile” feature of scale-free networks. From the perspective of attack information, random failure and intentional attack are merely two extremes in real-world networks. With the perfect attack information, one can remove the most important nodes preferentially according to some attack criteria, of which the most common is the degree of nodes. This attack strategy corresponds to the intentional attack. Without any attack information, one can only remove the nodes randomly. This attack strategy corresponds to the random failure. Information as an existence or expression format of thing’s movement is a common property of all matters. The robust-yet-fragile feature of scale-free networks reveals that the attack information can make an enormous difference of structural robustness. It inspires us to consider enhancing the structural robustness against intentional attacks by changing the displayed network structure information rather than modifying the network structure itself. If we can reduce the perfection of attack information by information disturbance, the critical hub nodes may survive during the intentional attacks and then the structural robustness of scale-free networks will be remarkably enhanced. To the best of our knowledge, this idea is new.

## Results

### Measuring the perfection of attack information

Consider networks formalized in terms of a simple undirected graph *G*(*V*, *E*), where *V* is the set of nodes and *E* is the set of edges. Denote by *N* = |*V*| the number of nodes. Denote by *k*_*i*_ the degree of node *v*_*i*_ and denote by *p*_*d*_(*k*) the degree distribution. If the degree distribution follows a power law, i.e., *p*_*d*_(*k*) = *ck* ^−*λ*^(*m* ≤ *k* ≤ *M*), where *m* is the minimum degree and *M* is the maximum degree of *G*, a network is called a scale-free network with the scaling exponent *λ*. The power-law distribution implies that nodes with only a few edges are numerous, but a very few nodes have a large number of edges. Due to the ubiquity of scale-free networks in the real-world, we focus on the structural robustness of scale-free networks in this study.

**Figure 1:**
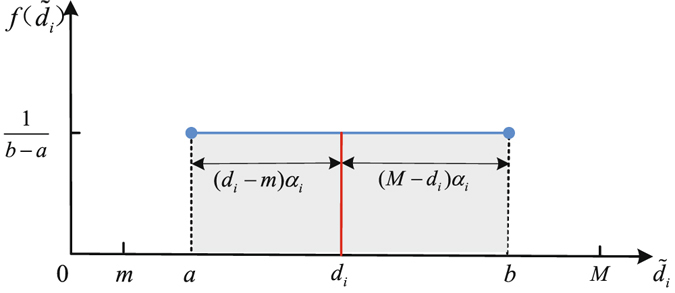
Illustration of the mathematical model of attack information. $f({\tilde{d}}_{i})$ is the probability density function of the displayed degree ${\tilde{d}}_{i}$. The information perfection parameter *α*_*i*_ characterizes the variability of the displayed degree ${\tilde{d}}_{i}$.

We only consider the node attack approaches in this study and assume that the attached edges are removed if one node is removed. We employ the degree of each node as the attack criterion, which means that the attacker will remove nodes in decreasing order of the degrees of nodes. We remark that the attack criterion has no essential effect on our model. Noting that the attacker may not obtain the perfect information, we denote by ${\tilde{d}}_{i}$ the displayed degree of a node *v*_*i*_ from the view of attacker and define it as the attack information. Although the true degree of a node is the objective existence, the displayed degree *d*_*i*_ will be generally different from the true degree *d*_*i*_. To measure the deviation from the displayed degree to the true degree, the displayed degree ${\tilde{d}}_{i}$ is supposed to spread out from the true degree towards the minimum node degree *m* and the maximum node degree *M* proportionately. For the purpose of convenience, we assume that the displayed degree ${\tilde{d}}_{i}$ follows a uniform distribution *U*(*a*, *b*) as shown in Fig. 1, where the minimum value

1 $$a={d}_{i}-({d}_{i}-m\mathrm{)(1}-{\alpha }_{i})={d}_{i}{\alpha }_{i}+m\mathrm{(1}-{\alpha }_{i})$$

and the maximum value

2 $$b={d}_{i}+(M-{d}_{i}\mathrm{)(1}-{\alpha }_{i})={d}_{i}{\alpha }_{i}+M\mathrm{(1}-{\alpha }_{i}\mathrm{)}.$$

Here, the perfection parameter of attack information *α*_*i*_ ∈ [0, 1] characterizes the variability of the displayed degree ${\tilde{d}}_{i}$. The larger *α*_*i*_ is, the narrower the distribution region is and then the more perfect the attack information is. There are two extreme cases. If *α*_*i*_ = 0 for all nodes, ${\tilde{d}}_{i}$ follows a uniform distribution in the region [*m*, *M*], which corresponds to the random failure. If *α*_*i*_ = 1 for all nodes, we obtain ${\tilde{d}}_{i}={d}_{i}$, which corresponds to the intentional attack.

It is easy to obtain that the expectation of the displayed degree of a node *v*_*i*_ is

3 $$E({\tilde{d}}_{i})=\frac{a+b}{2}={d}_{i}{\alpha }_{i}+\frac{(m+M\mathrm{)(1}-{\alpha }_{i})}{2}$$

and the standard deviation is

4 $$\sigma ({\tilde{d}}_{i})=\frac{(b-a)}{2\sqrt{3}}=\frac{(m+M\mathrm{)(1}-{\alpha }_{i})}{2\sqrt{3}}.$$

We remark that the fluctuation interval of the displayed degree ${\tilde{d}}_{i}$ of a node *v*_*i*_ may not be symmetrical around its true degree *d*_*i*_. If *d*_*i*_ < (*m* + *M*)/2, then $E({\tilde{d}}_{i})={d}_{i}{\alpha }_{i}+(m+M\mathrm{)(1}-{\alpha }_{i}\mathrm{)/2} > {d}_{i}$; if *d*_*i*_ > (*m* + *M*)/2, then $E({\tilde{d}}_{i})={d}_{i}{\alpha }_{i}+(m+M\mathrm{)(1}-{\alpha }_{i}\mathrm{)2} < {d}_{i}$. It suggests that the deviation from the displayed degree to the true degree may come from both the inaccuracy and the imprecision of attack information, where the accuracy refers to the closeness of agreement between a measurement and the true value, and the precision refers to the closeness of agreement of a set of measurements.

### Impact of attack information on structural robustness

**Figure 2:**
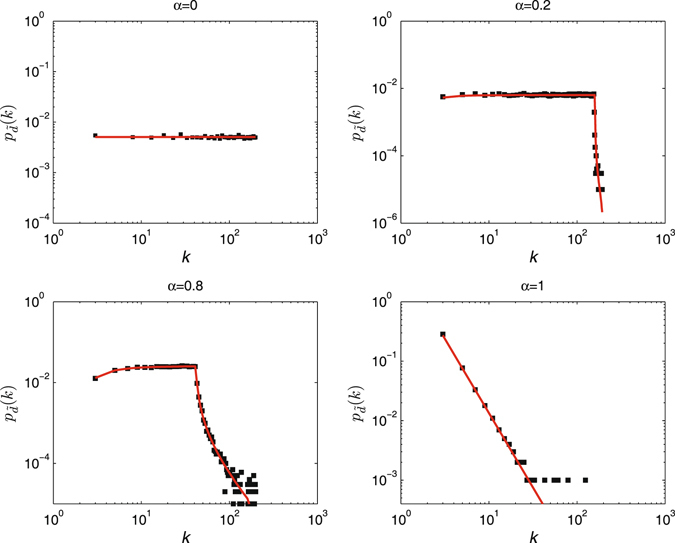
The displayed degree distribution (in a log-log scale) for various attack information perfection parameter *α* in a random scale-free network. The degree distribution follows *p*_*d*_(*k*) = (*λ* − 1)*m* ^*λ*−1^ *k* ^−*λ*^, where *N* = 1000, *λ* = 2.5 and *m* = 2. The simulation results are averaged over 100 independent realizations of imperfect attack information. The solid lines correspond to the analytical results (see Methods).

To explore the impact of attack information, we first show in Fig. 2 the displayed degree distribution ${p}_{\tilde{d}}(k)$ in random scale-free networks^20^, which is determined by the degree distribution *p*_*d*_(*k*) and the attack information perfection *α*_*i*_. For the purpose of convenience, let’s suppose that the attack information perfection *α*_*i*_ for all nodes is identical, i.e., *α*_*i*_ = *α*, *i* = 1, 2, …, *N*. We find that the displayed degree distributions gradually deviate from power-law distributions as the attack information perfection parameter *α* decreases.

**Figure 3:**
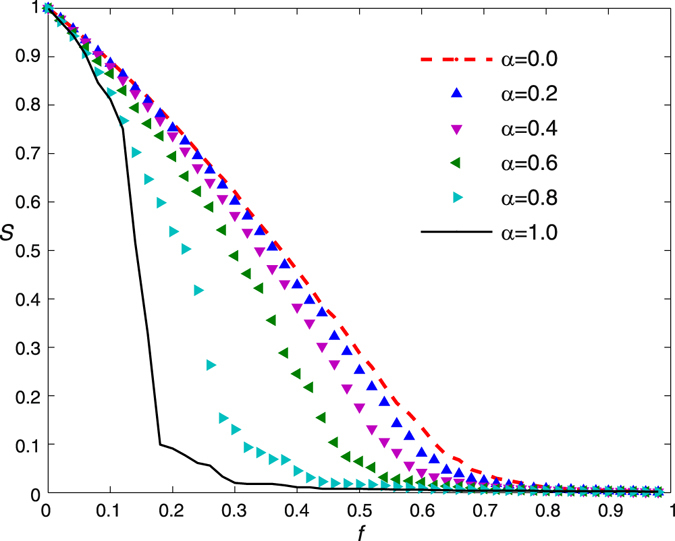
The relative sizes of the largest component *S* versus *f* for various attack information perfection parameter *α*. The original network is the same as the one we used in Fig. 2. The simulation results are averaged over 100 independent realizations of imperfect attack information.

We show the relative sizes of the largest component *S* as the removal fraction of nodes *f* increases for various attack information perfection parameter *α* in Fig. 3. It is easy to see that the attack information perfection has a considerable impact on *S*. If the attack information perfection parameter *α* is small, for example *α* = 0.2, the relative size of the largest component decreases slowly with the increasing of removal fraction of nodes and survives until a large fraction of the nodes are removed. However, if the attack information perfection parameter *α* is large, the *S* decreases abruptly at a small critical value of *f*.

**Figure 4:**
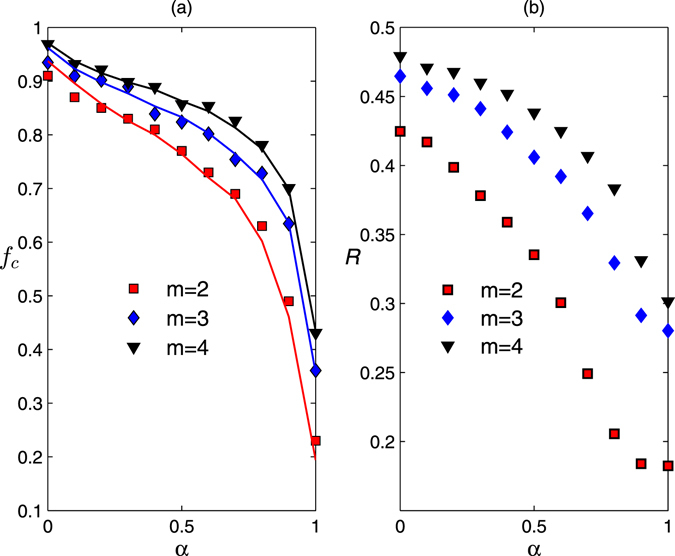
The critical removal fraction of nodes *f*_*c*_ (**a**) and network robustness *R* (**b**) versus attack information perfection parameter *α* in random scale-free networks. The degree distributions follow *p*_*d*_(*k*) = (*λ* − 1)*m* ^*λ*−1^ *k* ^−*λ*^, where *N* = 1000, *λ* = 2.5, *m* = 2 (■), *m* = 3 (◆) and *m* = 4 (▼). The simulation results are averaged over 100 independent realizations of imperfect attack information. The solid lines correspond to the analytical results.

Figure 4(a) shows the critical removal fraction *f*_*c*_ as a measure of network robustness^17^ both from the numerical and analytical results (see Methods). We observe that increasing the attack information perfection remarkably reduces the structural robustness of scale-free networks. From another perspective, we can enhance the structural robustness of scale-free networks by decreasing the perfection of attack information. For example, when *m* = 2, if we can decrease the attack information perfection parameter by information disturbance from *α* = 1 to *α* = 0.8, the critical removal fraction *f*_*c*_ can be increased from 23% to 63%. This means that information disturbance is an efficient strategy to enhance the structural robustness of scale-free networks. Moreover, we report the network robustness measure *R* ^21, 22^ as a function of the attack information perfection parameter *α* in Fig. 4(b) and observe the similar results as the case of *f*_*c*_. When *m* = 4, if we can decrease the attack information perfection by information disturbance from *α* = 1 to *α* = 0.8, the network robustness measure *R* can be increased from 0.3041 to 0.3834.

### Optimization model for disturbance strategies with cost constraint

In most realistic cases, we can not simultaneously disturbance all the nodes due to the cost constraint. Denote by *β*_*i*_ = 1 − *α*_*i*_ ∈ [0, 1] the disturbance strength parameter of a node *v*_*i*_, which represents the magnitude of information disturbance for the node *v*_*i*_. We call the vector *B* = [*β*_1_ *β*_2_ … *β*_*N*_] a disturbance strategy. We define a categorical variable *η*_*i*_ for each node *v*_*i*_ that *η*_*i*_ = 1 if the node *v*_*i*_ is disturbed, otherwise *η*_*i*_ = 0, i.e.,

5 $${\eta }_{i}=\{\begin{array}{cc}1 & {\rm{if}}\,{\beta }_{i} > 0\\ 0 & {\rm{if}}\,{\beta }_{i}=0\end{array}.$$

We denote by $\omega =\frac{1}{N}\sum _{i=1}^{N}\,{\eta }_{i}$ the disturbance range parameter. The case of *ω* = 1 has been studied in the previous section.

According to our mathematical model for attack information, the displayed degree of a node after information disturbance is a random variable rather than a definite value before information disturbance. We suppose that the uncertainty of the displayed degree of a node determines the of disturbance cost for it. The more uncertain the displayed degree of a node is, the costlier the disturbance strategy. We use the standard deviation of displayed degree of a node given in Eq. (4) to characterize the uncertainty of the displayed degree of a node and define the disturbance cost of a disturbance strategy as the sum of standard deviation of displayed degree for all nodes

6 $$C=\sum _{i=1}^{N}\,\sigma ({\tilde{d}}_{i})=\sum _{i=1}^{N}\,\frac{(m+M\mathrm{)(1}-{\alpha }_{i})}{2\sqrt{3}}=\frac{\sqrt{3}(m+M)}{6}\sum _{i=1}^{N}\,{\beta }_{i}.$$

Because 0 ≤ *β*_*i*_ ≤ 1, it is easy to obtain that the maximum disturbance cost is ${C}_{max}=\sqrt{3}(m+M)N\mathrm{/6}$ corresponding to the complete disturbance strategy *B*_*max*_ = [11…1]. In most cases, the disturbance cost is limited. We define the cost constraint as follows

7 $$\hat{C}={C}_{max}\cdot \theta =\frac{\sqrt{3}(m+M)N}{6}\theta ,$$

where *θ* ∈ [0, 1] is the cost constraint parameter. The larger the cost constraint parameter *θ* is, the more loose the constraint of disturbance cost is; the smaller the cost constraint parameter *θ* is, the tighter the constraint of disturbance cost is. In the extreme case of *θ* = 0, no nodes can be disturbed; in the extreme case of *θ* = 1, all nodes can be completely disturbed. The cost constraint condition $C\le \hat{C}$ leads to

8 $$\sum _{i=1}^{N}\,{\beta }_{i}\le N\cdot \theta .$$

Our goal is to enhance the structural robustness by choosing the disturbance strategy *B* = [*β*_1_ *β*_2_…*β*_*N*_] given the cost constraint parameter *θ*. Thus we define the effect of a disturbance strategy as

9 $${\rm{\Phi }}(B)=|{\rm{\Gamma }}(B)|,$$

where Γ is the structural robustness measure, such as *f*_*c*_ or *R* (see Methods), and |Γ(*B*)| represents the expectation of the structural robustness measure under a disturbance strategy *B*. Thus, the optimization model of disturbance strategies can be described as follows

10 $$\begin{array}{c}{\rm{\max }}\,{\rm{\Phi }}(B=[{\beta }_{1}{\beta }_{2}\ldots {\beta }_{N}])\\ {\rm{s}}{\rm{.t}}.\{\begin{array}{c}\sum _{i=1}^{N}\,{\beta }_{i}\le N\cdot \theta \\ 0\le {\beta }_{i}\le 1\end{array}.\end{array}$$

For large *N*, it will be a large-scale optimization problem^23^, which is very time-consuming. For the convenience of analysis, we next consider a simplified version of the optimization model presented above. We assume that the disturbance strength parameters *β*_*i*_ for all the disturbed nodes (*η*_*i*_ = 1) are identical, i.e.,

11 $${\beta }_{i}=\beta \cdot {\eta }_{i}.$$

where *β* ∈ (0, 1]. Thus the disturbance cost can be written as

12 $$C=\frac{\sqrt{3}(m+M)}{6}\sum _{i=1}^{N}\,{\beta }_{i}=\frac{\sqrt{3}(m+M)\beta }{6}\sum _{i=1}^{N}\,{\eta }_{i},$$

and then the optimization model for disturbance strategies can be transformed into

13 $$\begin{array}{l}{\rm{\max }}\,{\rm{\Phi }}(B=\beta \cdot [{\eta }_{1}{\eta }_{2}\ldots {\eta }_{N}])\\ {\rm{s}}{\rm{.t}}.\{\begin{array}{l}\beta \cdot \sum _{i=1}^{N}\,{\eta }_{i}\le N\cdot \theta \\ {\eta }_{i}=\mathrm{0,}\,1\\ 0 < \beta \le 1\end{array}.\end{array}$$

It means that the optimization problem is just simplified to determine the node set to disturb and the magnitude of information disturbance.

According to the analysis above, we know that the disturbance effect Φ decreases monotonically as the attack information perfection parameter *α* increases and hence increases monotonically as the disturbance strength parameter *β* increases. Therefore, to maximize Φ, the parameter *β* should take the maximum value under the conditions of constraint $\beta \cdot \sum _{i=1}^{N}\,{\eta }_{i}\le N\cdot \theta$ and 0 < *β* ≤ 1, which leads to

14 $$\beta =\{\begin{array}{cc}N\cdot \theta /\sum _{i=1}^{N}\,{\eta }_{i} & {\rm{if}}\,\sum _{i=1}^{N}\,{\eta }_{i} > N\cdot \theta \\ 1 & {\rm{if}}\,\sum _{i\mathrm{=1}}^{N}\,{\eta }_{i}\le N\cdot \theta \end{array}.$$

Noting that $\sum _{i=1}^{N}\,{\eta }_{i}=N\cdot \omega$, then we obtain that

15 $$\beta =\{\begin{array}{cc}\theta /\omega  & {\rm{if}}\,\,\omega  > \theta \\ 1 & {\rm{if}}\,\,\omega \le \theta \end{array}\,{\rm{or}}\,\beta =\,{\rm{\min }}\{\theta /\omega ,\,1\}.$$

Thus the optimization model for disturbance strategies presented in Eq. (13) can be written as

16 $$\begin{array}{l}{\rm{\max }}\,{\rm{\Phi }}(B=\,{\rm{\min }}\{\theta /\omega \mathrm{,1}\}\cdot [{\eta }_{1}{\eta }_{2}\ldots {\eta }_{N}])\\ {\rm{s}}{\rm{.t}}.{\eta }_{i}=\mathrm{0,}\,1\end{array}$$

It means that the optimization problem is just simplified to determine how many and which nodes should be disturbed.

To determine which nodes are disturbed, we transform the process of determining into an unequal probability sampling problem without replacement. We define the selection probability that a node *v*_*i*_ is sampled to disturb in each sample as follows

17 $${\nabla }_{i}=\frac{{{d}_{i}}^{\delta }}{{\sum }_{t=1}^{N}\,{{d}_{t}}^{\delta }},$$

where *δ* ∈ [−1, 1] is the disturbance strategic parameter. If *δ* > 0, the high-degree nodes are disturbed preferentially; if *δ* < 0, the low-degree nodes are disturbed preferentially; if *δ* = 0, the nodes are disturbed randomly. Consequently, the simplified version of optimization model for disturbance strategies can be described as follows

18 $$\begin{array}{l}{\rm{\max }}\,{\rm{\Phi }}(B={\rm{\Theta }}(\omega ,\delta ))\\ {\rm{s}}{\rm{.t}}.\{\begin{array}{c}0\le \omega \le 1\\ -1\le \delta \le 1\end{array}\end{array},$$

where Θ:(*ω*, *δ*) → *B* corresponds to the procedure of unequal probability sampling.

### Optimal disturbance strategies for enhancing structural robustness

**Figure 5:**
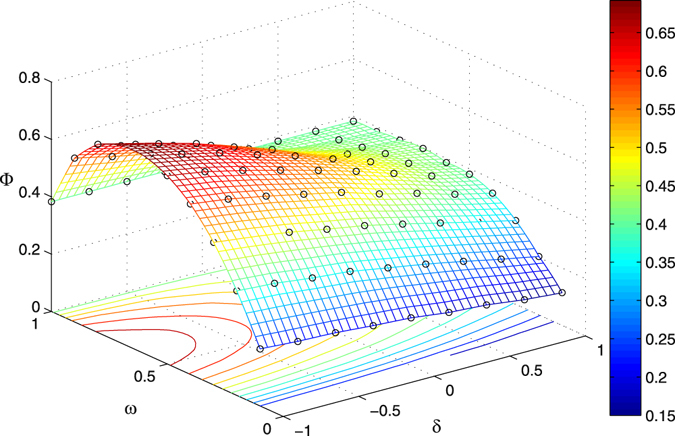
The Kriging-based response surface and contour plots for the disturbance effect. The original network is the same as the one we used in Fig. 2. The cost constraint parameter *θ* is 0.1. The circles represent the original experiment data, which are averaged over 100 realizations of disturbance strategy. The disturbance effect is obtained based on the structural robustness measure *R*.

We next investigate the optimal disturbance strategies for enhancing structural robustness based on the simplified optimization model in Eq. (18). Because the calculation of structural robustness measure Γ might be computationally expensive, and furthermore, we need to take the average over many realizations of disturbance strategy to evaluate the disturbance effect Φ, the exploration of the solution space are largely limited. Therefore, we consider to use the statistical approximations to construct a meta-model, which provides a surrogate model of the original optimization problem. The surrogate model can be estimated from experiment data by running the simulation experiments on a sample of points in the region of interest. We employ the popular Kriging surrogate models^24^ in this study. We show the Kriging-based response surface and contour plots for the disturbance effect in Fig. 5.

**Figure 6:**
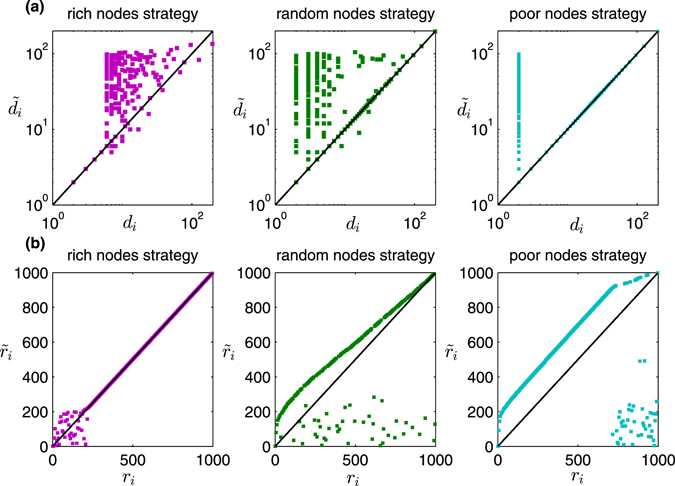
Degree pairs (**a**) and rank pairs (**b**) of nodes before and after information disturbance under three typical disturbance strategies. The original network is the same as the one we used in Fig. 2. The cost constraint parameter *θ* is 0.1 and the disturbance range parameter *ω* is 20%. The solid lines are the reference lines which represent that ${d}_{i}={\tilde{d}}_{i}\,{\rm{or}}\,{r}_{i}={\tilde{r}}_{i}$.

From the response surface and the contour plots in Fig. 5, we surprisingly find that the disturbance effect decreases monotonically with the increase of disturbance strategic parameter *δ* given the disturbance range parameter *ω*. We observe similar results in scale-free networks with various parameters. It suggests that, given the disturbance range parameter *ω*, disturbing the “poor nodes” with low degrees may be more effective in scale-free networks, i.e., the optimal disturbance strategic parameter is *δ* ^*^ = −1. This observation is counterintuitive but interesting. To explain this phenomenon, we sort all nodes in decreasing order of their degrees *d*_*i*_ before information disturbance and their displayed degrees ${\tilde{d}}_{i}$ after information disturbance, respectively. Denote by *r*_*i*_ and ${\tilde{r}}_{i}$ the rank of *v*_*i*_ before and after information disturbance, respectively. In Fig. 6, we plot the degree pairs $({d}_{i},{\tilde{d}}_{i})$ and the rank pairs $({r}_{i},{\tilde{r}}_{i})$ under three typical disturbance strategies: (1) “rich nodes strategy” with *δ* = 1; (2) “random nodes strategy” with *δ* = 0; (3) “poor nodes strategy” with *δ* = −1. Under the “rich nodes strategy”, we observe that the degrees of high-degree nodes change a lot after information disturbance, but their ranks change very little and then the small “rich group” remains basically unchanged. It leads to the fact that high-degree nodes will still be removed after information disturbance. However, under the “poor nodes strategy”, we observe that both the degrees and the ranks of low-degree nodes change a lot after information disturbance and then many “poor nodes” infiltrate the “rich group” after information disturbance. Thus, the high-degree nodes can survive during the attack. These findings suggest that what really matters to the enhancement of structural robustness is the change of ranks of nodes rather than the change of the degrees of nodes itself. Due to the heterogeneous degree distributions, the “poor nodes strategy” disturbs the ranks of nodes in scale-free networks more dramatically and then is more effective than the “rich nodes strategy”.

**Figure 7:**
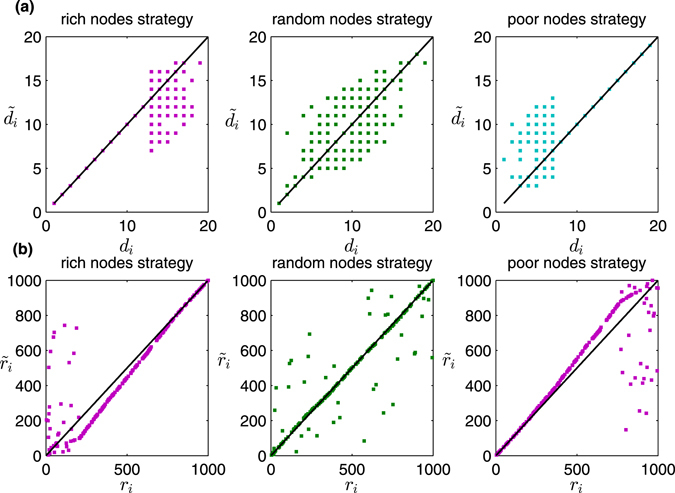
Degrees (**a**) and ranks (**b**) of nodes before and after information disturbance under three typical disturbance strategies. The original network is an ER random graph *G*_*N*,*p*_, where *N* = 1000 and *q* = 0.01. The cost constraint parameter *θ* is 0.1 and the disturbance range parameter *ω* is 20%. The solid lines are the reference lines which represent that ${d}_{i}={\tilde{d}}_{i}\,{\rm{or}}\,{r}_{i}={\tilde{r}}_{i}$.

To verify this judgment, we implement similar experiments in Erdös-Rényi (ER) random graphs^25^ and show the results in Fig. 7. The ER random graph *G*_*N*,*p*_ is obtained by starting with a set of *N* nodes and adding edges between them at random such that each of the possible *N*(*N* − 1)/2 edges occurs independently with probability *p*. The ER random graph has a Poisson degree distributions for large *N* such that most nodes in the network have similar degrees. In contrast to the case of scale-free networks, we find that the “rich nodes strategy” seems to be more effective than the “poor nodes strategy” in ER random graphs. This result can also be explained by our observation that many high-degree nodes can survive after information disturbance under the “rich nodes strategy”, while the “rich group” remains almost unchanged under the “poor nodes strategy”.

**Figure 8:**
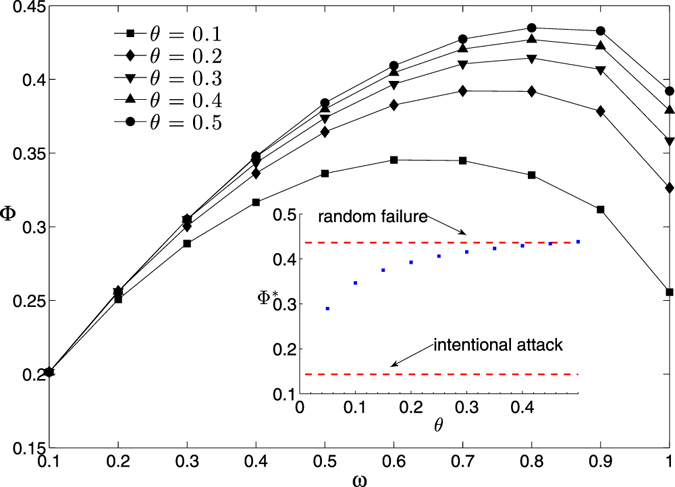
The disturbance effect Φ versus the disturbance range parameter *ω* under the “poor nodes strategy”. The original network is the same as the one we used in Fig. 2. The symbols represent the original experiment data, which are averaged over 100 realizations of disturbance strategy. The solid lines are obtained from the Kriging surrogate model. The insert shows the maximum disturbance effect Φ^*^ as a function of the cost constraint parameter *θ* and the cases of random failure and intentional attack as references (dotted lines). The disturbance effect is obtained based on the structural robustness measure *R*.

We next investigate the optimal disturbance range parameter *ω* for enhancing structural robustness of scale-free networks. We show in Fig. 8 the relationship between the disturbance effect Φ and the disturbance range parameter *ω* under the “poor nodes strategy” (*δ* ^*^ = −1). We observe that the disturbance effect Φ achieves a maximum value Φ^*^ (see the inset in Fig. 8) at the optimal disturbance range parameter *ω* ^*^. We find that, even with a very small cost constraint parameter *θ* = 0.05, the maximum disturbance effect Φ^*^ can be increased from 0.1433 (intentional attack without information disturbance) to 0.2897. In other words, disturbance with an average disturbance strength parameter 0.05 can almost double the structural robustness of scale-free networks. Moreover, we observe that, if the cost constraint parameter *θ* increases to 0.5, the maximum disturbance effect Φ^*^ can achieve to the case of random failure (0.4361).

**Figure 9:**
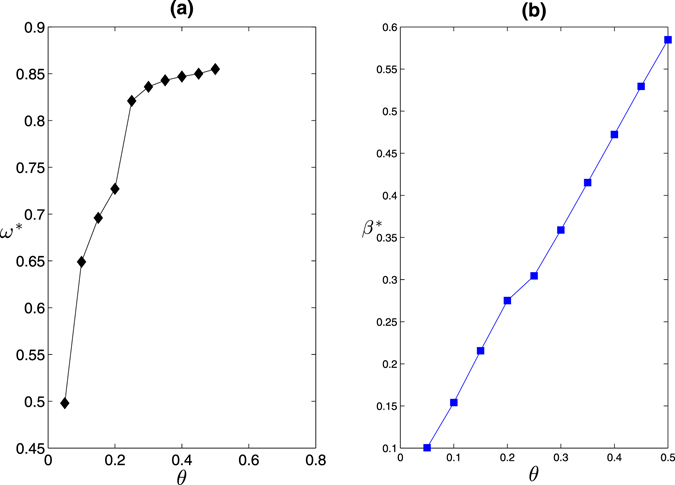
The optimal disturbance range parameter *ω* ^*^ (**a**) and the optimal disturbance strength parameter *β* ^*^ (**b**) versus the cost constraint parameter *θ*. The original network is the same as the one we used in Fig. 2. The disturbance effect is obtained based on the structural robustness measure *R*.

We show in Fig. 9 the optimal disturbance range parameter *ω* ^*^ and the corresponding optimal disturbance strength parameter *β* ^*^ based on Eq. (15) as a function of the cost constraint parameter *θ*. We can obtain the optimal disturbance strategies with various cost constraint parameters *θ*. For example, with *θ* = 0.05, we should disturb about 50% nodes with the disturbance strength parameter 0.1. We find that the optimal disturbance range parameter *ω* ^*^ firstly increase rapidly as the cost constraint parameter *θ* increases and then achieve to a stable value when *θ* is large. Moreover, we find that the optimal disturbance strength parameter *β* ^*^ increase approximately linearly as the cost constraint parameter *θ* increases.

### Comparison with other approach for enhancing structural robustness

**Figure 10:**
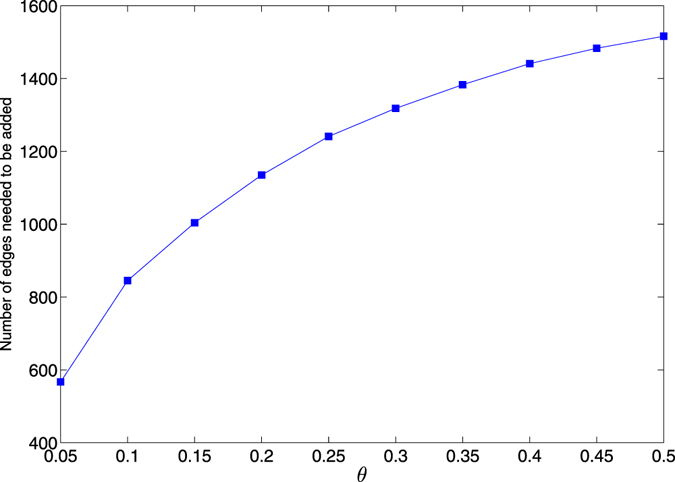
The edge addition method versus the information disturbance method. The original network is the same as the one we used in Fig. 2. The disturbance effect is obtained based on the structural robustness measure *R*.

To furthermore demonstrate the efficiency of our method, we next compare our method with the edge addition method. In each step of the edge addition method, we add randomly one nonexistent edge to enhance the structural robustness. We show in Fig. 10 the number of edges needed to be added to achieve the same structural robustness measure with various cost constraint parameters *θ*. We find that we need to add near 600 edges to achieve the same effect even with the small cost constraint parameter *θ* = 0.05. It indicates that enhancing structural robustness of scale-free networks against intentional attacks by information disturbance is a cost-efficient approach.

### Experiments in real-world networks

Modern society is dependent on well-functioning infrastructure. To valid the feasibility of our method, we consider two of the most fragile, but critical infrastructures: the power grid and the fiber network. The breakdown of any of these networks would constitute a major disaster due to the strong dependency of modern society on energy and information. We here apply our method to the India power grid^26^ consisting of *N* = 572 nodes and *W* = 871 edges and the fiber backbone operated by a major U.S. network provider (CenturyLink) consisting of *N* = 154 nodes and *W* = 206 edges. Both networks have power-law degree distributions.

We first calculate the original structural robustness measure *R* ^0^ for the India power grid (IPG) and the U.S. fiber network (USF). It is obtained that ${R}_{IPG}^{0}=0.0912$ and ${R}_{USF}^{0}=0.1341$. We then use the method introduced above to explore the optimal disturbance strategies with various cost-constrain parameters. The results are shown in Table 1. It is easy to find that the structural robustness of both IPG and USF is dramatically enhanced by the information disturbance. For example, with the cost constraint parameter *θ* = 0.3, the optimal disturbance strategy can almost double the structural robustness of IPG from ${R}_{IPG}^{0}=0.09$ to Φ^*^ = 0.1801. Moreover, we see that, for both IPG and USF, the optimal disturbance strategy is try to disturb intensively the “poor nodes” with low degrees (*δ* ^*^ = −1) with high disturbance strength parameter (*β* ^*^ ≈ 1).

**Table 1:** The optimal disturbance strategies in two real-world critical infrastructure networks.

Networks	*θ* = 0.1				*θ* = 0.2				*θ* = 0.3			
	*δ* ^*^	*ω* ^*^	*β* ^*^	Φ^*^	*δ* ^*^	*ω* ^*^	*β* ^*^	Φ^*^	*δ* ^*^	*ω* ^*^	*β* ^*^	Φ^*^
IPG	−1	0.10	1	0.1245	−1	0.20	1	0.1548	−1	0.30	1	0.1801
USF	−1	0.11	0.9091	0.1605	−1	0.20	1	0.1799	−1	0.31	0.9677	0.1976

IPG represents the India power grid and USF represents the U.S. fiber network.

## Discussions

Many real-world systems can be described by scale-free networks with power-law degree distributions, leading to the “robust yet fragile” feature. The enhancement of structural robustness of scale-free networks against intentional attack is an important and challenging problem. In this study, we assumed that the attacker removes nodes according to some attack criteria, of which the most common is the degree of nodes. Then the attack criterion can be considered as the attack information. With perfect attack information, one can preferentially remove the most important nodes in the network (intentional attack). However, in many realistic settings, the attacker can not obtain perfect attack information. For example, the displayed degree of nodes from the view of the attacker may deviate from the true degree. Thus, the attacker wants to gain the attack information as much as possible to destroy a network using various reconnaissance means, whereas the defenders want to conceal the attack information as much as possible to protect a network using various disturbance means. It inspires us to consider enhancing the structural robustness against intentional attacks by changing the displayed network structure information rather than modifying the network structure itself.

We first introduced a mathematical model for attack information based on the node degree. Instead of a certain value, we assumed that the displayed degree ${\tilde{d}}_{i}$ with imperfect attack information is a random variable following a uniform distribution, which can be controlled by a normalized perfection parameter *α*_*i*_ ∈ [0,1]. With this assumption, we can derive analytically the displayed degree distribution and the measure of structural robustness with the imperfect attack information. It is worth mentioning that, even we use degree in the model, the method can be readily extended to other attack criteria, such as node betweenness, closeness, etc. We investigated the impact of attack information on the structural robustness of scale-free networks both analytically and numerically. It was shown that reducing the attack information perfection by information disturbance can dramatically enhance the structural robustness of scale-free networks. We then proposed a generalized and simplified optimization model of disturbance strategies. In the simplified optimization model, it is assumed that the disturbance strength parameter for all the disturbed nodes is identical. We solved the simplified optimization model based on the Kriging-based response surface. Intuitively, one should disturb “rich nodes” with high degrees preferentially to enhance the structural robustness. But we found with surprise that, with cost constraints, disturbing “poor nodes” with low degrees is more effective in scale-free networks. We explained this counterintuitive phenomenon by comparing with Erdös-Rényi (ER) random graphs and emphasized that it stems from the heterogeneous degree distributions of scale-free networks. It is worth noting that this finding is based on the assumption of identical disturbance strength parameters. The optimal disturbance strategy with non-identical disturbance strength parameters remains an open and challenging problem. Lastly, we demonstrated the efficiency of our method by comparing with the edge addition method and validate the feasibility of our method in two real-world critical infrastructure networks, i.e., the India power grid and the U.S. fiber network. Although our results can not apply to all networks with various degree distributions, it is of great significance because of the ubiquity of scale-free networks.

It is intuitive to enhance structural robustness of scale-free networks against intentional attacks by information disturbance. The fundamental objective of this approach is to make important nodes less distinguishable with less important nodes by information disturbance, and thus the important nodes can survive during the intentional attacks. In most cases, changing the displayed information of network structure is easier and more realistic than reconstructing the network. Although we didn’t give the practical disturbance scheme from a technical perspective, the main contribution of this paper is to present a general theoretical framework and demonstrate the efficiency and the feasibility of this method from a methodological perspective. We believe that our model and findings may lead to useful insights on developing effective attack or defense strategies in scale-free networks, so as to protect infrastructure systems (e.g., power grids, telecommunications, transportation and water-supply systems) against terrorist attacks, or reduce undesired highly synchronized behavior in the central nervous system (e.g., Parkinson’s disease, epilepsy, and other pathological rhythmic activities), etc.

## Methods

### Measures of structural robustness with imperfect attack information

Simple and effective measures of structural robustness are essential for the study of network resistance. We first consider the critical removal fraction of nodes to characterize the structural robustness of scale-free networks with imperfect attack information^17, 27^. It characterizes statistically how the removal of nodes leads to the disintegration of the network at a given critical removal fraction *f*_*c*_. The larger the *f*_*c*_ is, the more robust the network is. The disintegration of a network is measured in terms of network performance, which is characterized most often with the size of the largest component. In this study, we choose *κ* ≡ <*k* ^2^>/<*k>* <2 as the criterion for determing the disintegration of a network^28, 29^. After each node is removed, we calculate *κ*. When *κ* becomes less than 2, we record the number of nodes *t* removed up to that point. The threshold *f*_*c*_ is obtained as *f*_*c*_ = <*t*>/*N*.

Furthermore, because the structural robustness measure *f*_*c*_ is the critical fraction of attacks at which the network completely collapses, it ignores situations in which the network suffers a big damage without completely collapsing. To demonstrate the impact of attack information in depth, we also consider the network robustness measure *R* defined as^21, 22^

19 $$R=\frac{1}{N+1}\sum _{Q=0}^{N}\,S(Q)$$

where *S*(*Q*) is the fraction of nodes in the largest component after removing *Q* = *Nf* nodes in decreasing order of the displayed node degree $\tilde{d}$. The normalization factor 1/(*N* + 1) ensures that the network robustness with different sizes can be compared. The network robustness *R*, which corresponds to the integral of the curves *S*(*Q*), not only measures after how many removals the network collapse, but also considers the size of the largest component for each number of removed nodes. The range of possible *R* values is between 1/(*N* + 1) and 0.5, where *R* = 0 corresponds to an empty network of isolated nodes and *R* = 0.5 corresponds a fully connected network.

### The displayed degree distribution with imperfect attack information

For the purpose of convenience, let’s suppose that the attack information perfection parameter *α*_*i*_ for all nodes is identical, i.e., *α*_*i*_ = *α*, *i* = 1, 2, …, *N*. Considering that the displayed degree ${\tilde{d}}_{i}$ follows a uniform distribution *U*(*a*, *b*), we first formalize ${\tilde{d}}_{i}$ as

20 $$\begin{array}{rcl}{\tilde{d}}_{i} & = & a+(b-a)u\\  & = & {d}_{i}\alpha +m\mathrm{(1}-\alpha )+(M-m\mathrm{)(1}-\alpha )u,\end{array}$$

where *u* is a random variable which follows a uniform distribution over the unit interval [0, 1]. Let Ψ = *d*_*i*_ *α* and Δ = *m*(1 − *α*) + (*M* − *m*)(1 − *α*)*u*, then we obtain that

21 $${\tilde{d}}_{i}={\rm{\Psi }}+{\rm{\Delta }}.$$

Noting that

22 $${p}_{d}(k)=\{\begin{array}{cc}c{k}^{-\lambda } & m\le k\le M\\ 0 & {\rm{others}}\end{array},$$

where *c* ≈ (*λ* − 1)*m* ^*λ*−1^ and *M* ≈ *mN* ^1/(*λ*−1)^ ^30^. We then obtain

23 $${p}_{{\rm{\Psi }}}(k)=\{\begin{array}{cc}c^{\prime} {k}^{-\lambda } & m\alpha \le k\le M\alpha \\ 0 & {\rm{others}}\end{array},$$

where *c*′ = *α* ^*λ*−1^(*λ* − 1)*m* ^*λ*−1^. Moreover, it is easy to obtain

24 $${p}_{{\rm{\Delta }}}(k)=\{\begin{array}{cc}\mathrm{1/(}M-m\mathrm{)(1}-\alpha ) & m\mathrm{(1}-\alpha )\le k\le M\mathrm{(1}-\alpha )\\ 0 & {\rm{others}}\end{array}.$$

Consequently, we can obtain the probability distribution of displayed degree $\tilde{d}$ using the convolution formula

25 $${p}_{\tilde{d}}(k)={\int }_{t=-\infty }^{t=+\infty }{p}_{{\rm{\Psi }}}(t){p}_{{\rm{\Delta }}}(k-t)dt={\int }_{t=m\alpha }^{t=M\alpha }{p}_{{\rm{\Psi }}}(t){p}_{{\rm{\Delta }}}(k-t)dt.$$

### The critical removal fraction with imperfect attack information

In this study, we use the generating function formalism^31, 32^ to derive the critical removal fraction *f*_*c*_ in random scale-free networks. We first sort all nodes in decreasing order of $\tilde{d}$. Denote by $r(\tilde{k})$ the rank of a node with the displayed degree $\tilde{k}$. Denote by $\tilde{K}$ the maximum displayed degree among the remaining nodes after a fraction *f* of nodes are removed in decreasing order of $\tilde{d}$. Noting that

26 $$r(\mathop{K}\limits^{\sim })=N\sum _{k=\tilde{K}+1}^{M}\,{p}_{\tilde{d}}(k)=Nf.$$

Then $\tilde{K}$ can be obtained by solving Eq. (26).

Denote by *q*(*k*) the probability that a node with degree *k* is not removed. It equals to the probability that the displayed degree $\tilde{d}$ of a node with degree *k* is not larger than than $\tilde{K}$. Considering that $\tilde{d}$ is stochastic and follows the uniform distribution in the region [*kα* + *m*(1 − *α*), *kα* + *M*(1 − *α*)]. Then we can formalize *q*(*k*) as

27 $$q(k)=\{\begin{array}{cc}0 & \tilde{K} < k\alpha +m\mathrm{(1}-\alpha )\\ \frac{\tilde{K}-k\alpha -m\mathrm{(1}-\alpha )}{(M-m\mathrm{)(1}-\alpha )} & k\alpha +m\mathrm{(1}-\alpha )\le \tilde{K}\le k\alpha +M\mathrm{(1}-\alpha )\\ 1 & \tilde{K} > k\alpha +M\mathrm{(1}-\alpha )\end{array}$$

Noting that a giant component forms under the critical condition^28^

28 $$\frac{{\sum }_{k}\,k(k-\mathrm{1)}p(k)q(k)}{{\sum }_{k}\,kp(k)}=1.$$

Substituting Eq. (27) into Eq. (28), we can solve the critical removal fraction *f*_*c*_.
